# 

**Published:** 1880-12-20

**Authors:** 


### TABLE OF CONTENTS.

ORIGINAL AND SELECTED ARTICLES.

Typhoid Fever, by J. H. Egan, M.D., Pulaski, Tenn., 433. Tic Douloureux of Twelve Months' Standing Cured by Resection of the InfrarOrbital Nerve and its Branches, by R. S. Jenkins, M.D., of Georgia, 438. Some of the Therapeutic Effects of Hydrochlorate of Ammonia, by Thomas S. Mitchell, M.D., of Georgia, 439. A Case in Practice, by J. Hendree, of Alabama, 440. Pilocarpin in the Treatment of Malarial Fever, 441. The Telephone and Microphone in Auslultation, 444. Prolapse of the Ovaries—Pelvic Hæmatocele, 448.

ABSTRACTS AND GLEANINGS.

Prolapsus Ani in Children; Vaccine Virus, 451. Chronic Lung Diseases, 452. Withdrawing Fluids from the Middle Ear; Clover Tea for Cancer; Tea as an Antidote to Opium, 453. Points in the Surgery of the Urinary Organs, 454. Action of Sugar on Calomel, 455. Hypodermic Use of Quinine; Erythoxylon Coca, 456. Treatment of Scarlet Fever by Warm Baths, 457. Hot Rectal Douche, 458. Ulceration of the Bowel; Death of Miss Adelaide Neilson; Aihun, 459. The Two Bogus Colleges Finally Wiped Out; Ovariotomy; Ingrowing Nails, 460. Intermittents; Consumption; Fasting or Starvation, 461. Topical Use of Ergot; Quinine as an Anti-Abortive; Summer Complaint; Beri-Beri; Treatment of Puerperal Fever, 462.

SCIENTIFIC ITEMS.

Copying Ink; Why the Needle Points to the North; Lunar Geology; An Electro-Magnetic Experiment, 463. The Blood of Intermittents; A New Battery; A New Lens; Atoms, 464.

PRACTICAL NOTES AND FORMULÆ.

Dyspepsia in Infants; Pruritus Vulvæ, 465. Desiccated Blood; Disturbed Menstruation; Remedy for Corns; Turkish Bath in Hay Asthma, 466. Sore Nipples; Preservative Fluid; Falling of the Hair; Only a Slight Gonorrhœa, 467. Painful Hemorrhoids; Gaseous Dyspepsia; Hemorrhoids; Diphtheria; Tubercular Laryngitis, 478.

EDITORIAL AND MISCELLANEOUS.

Special Notice; Editorial Notices, 469. Our Journal Next Year; Arrearages—How to "Feel Better;" Book Notices, 470.

